# Heatstroke-Induced Inflammatory Response and Therapeutic Biomarkers

**DOI:** 10.3390/biomedicines13020261

**Published:** 2025-01-21

**Authors:** Piyush Baindara, Aritra Jana, Roy Dinata, Santi M. Mandal

**Affiliations:** 1 Animal Sciences Research Center, Division of Animal Sciences, University of Missouri, Columbia, MO 65201, USA; 2 Whitney M. Young Magnet High School Chicago, Chicago, IL 60607, USA; aritraj8@gmail.com; 3 Department of Biological Sciences, Indian Institute of Science Education and Research, Kolkata 741246, India; dinataroy9@gmail.com; 4 Department of Chemistry and Biochemistry, University of California San Diego, San Diego, CA 92093, USA; mandalsm@gmail.com

**Keywords:** heatstroke, inflammation, neuroinflammation, hyperthermia, multiorgan failure, biomarkers

## Abstract

In recent years, heatstroke has become one of the most dangerous illnesses associated with hyperthermia. Hyperthermia is described as an increased body temperature, where there is more heat accrual than dissipation, which happens during environmental heat stress conditions or exhaustive exercise and subsequently leads to heatstroke. Heatstroke is characterized as a dysfunction of the central nervous system (CNS), associated with neuroinflammation, including utmost hyperthermia, which eventually leads to multiorgan failure. Heatstroke-related fatalities have rapidly increased in the recent past; however, there is still a gap in the understanding of heatstroke and associated outcomes during heatstroke. Especially of note, early diagnosis of heatstroke-related complications is one of the important aspects that need to be addressed. This article reviewed current knowledge about heatstroke and associated inflammatory responses, including neuroinflammation and other clinical complications. Using molecular dynamics simulation analysis of triose phosphate isomerase (a housekeeping enzyme) at different temperatures, we demonstrated how protein structures, and thus their functions, can be varied with temperature increases. Additionally, we discussed therapeutically relevant biomarkers of heatstroke which might be helpful in the early detection of heatstroke possibilities and candidate drug targets to control or minimize heatstroke events.

## 1. Introduction

**Figure 1:**
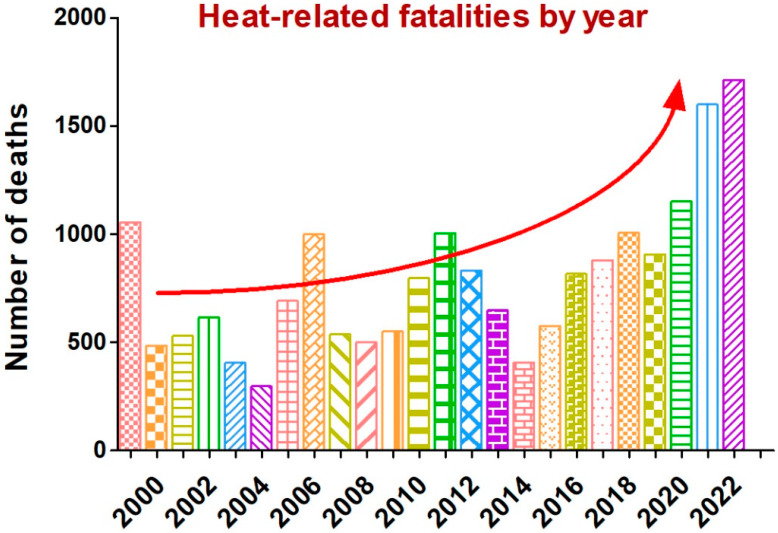
Heat-related fatalities in the United States from the year 1999 to 2022. Data acquired from the Center for Disease Control and Prevention, United States (Accessed on 7 November 2024). The red curved arrow indicates the increase in deaths per year.

Heatstroke is one of the most severe conditions in which core body temperature is overly enhanced when heat accumulation exceeds heat dissipation during excess exercise or extreme environmental heat stress. In general, the clinical symptoms of heatstroke include severe hyperthermia (typically >40.5 °C), dysfunction of the CNS, and multiorgan failure [1,2]. Heatstroke is one of the most severe or fatal conditions out of all heat-related illnesses. Deaths caused by heatstroke are among the top three mortality causes, especially in athletes, and are estimated to rise at least 2.5-fold in the next 30 years [3,4]. Heatstroke leads to ultimate death by affecting many systems, through coagulopathy, respiratory failure, liver and kidney failure, along with gastrointestinal disorders; however, the death rate is influenced by the severity of organ dysfunction along with the total duration of hyperthermia [5]. A death rate of approximately 80% has been estimated if heatstroke is not treated; however, this remains above 60% even after critical care [5,6]. In the United States alone, there were 1714 heat-related fatalities recorded in the year 2022 (Figure 1). Reportedly, male fatalities are higher than females, with the highest rate in the age group being 55–64 (https://usafacts.org/articles/how-many-people-die-from-extreme-heat-in-the-us, accessed on 7 November 2024). Although heatstroke symptoms and illness conditions need to be understood in detail, early identification of patients in prehospital conditions, emergency medical or critical care teams, and starting immediate treatment are always helpful. This review focused on the heatstroke-associated inflammatory response, including neuroinflammation, clinical symptoms, biomarkers, and prevention strategies.

## 2. Heatstroke Definition

Heatstroke was first defined by Bouchama and Knochel as a CNS dysfunction, delirium, convulsions, or coma, associated with a core body temperature of more than 40 °C, and is the most extensively used and well-known definition of heatstroke [7]. Another well-known definition of heatstroke is provided by the Japanese Association for Acute Medicine (JAAM) which states heatstroke is a high-environmental-temperature-associated illness along with either disseminated intravascular coagulopathy, CNS dysfunction, or haptic or renal abnormalities, and body temperature is not included as a diagnostic parameter [1]. Further, heatstroke is divided into two subtypes, classic heatstroke and exertional heatstroke. Classic heatstroke is defined to be caused by passive heat exposure with inadequate heat dissipation mechanisms. People who are deemed more vulnerable, such as elderly people with underlying medical issues linked to a decreased capacity for thermoregulation, or children who are not yet pubescent, often experience classic heatstroke during heat waves or exposure to high environmental temperatures [8,9,10]. On the other hand, exertional heatstroke usually happens to normally healthy people who engage in physically demanding activities, such as military services or endurance sports, as well as some professional workers, like fire and police officers. Most importantly, as skeletal muscle action generates a lot of heat in exertional heatstroke compared to classic heatstroke, it might occur at low ambient temperatures as well [11,12]. Although heatstroke is well defined in general, further detailed studies at the molecular level are required to determine the associated biomarkers for the diagnosis of early onset and rescue of the associated mortalities.

## 3. Pathogenesis of Heatstroke

**Figure 2:**
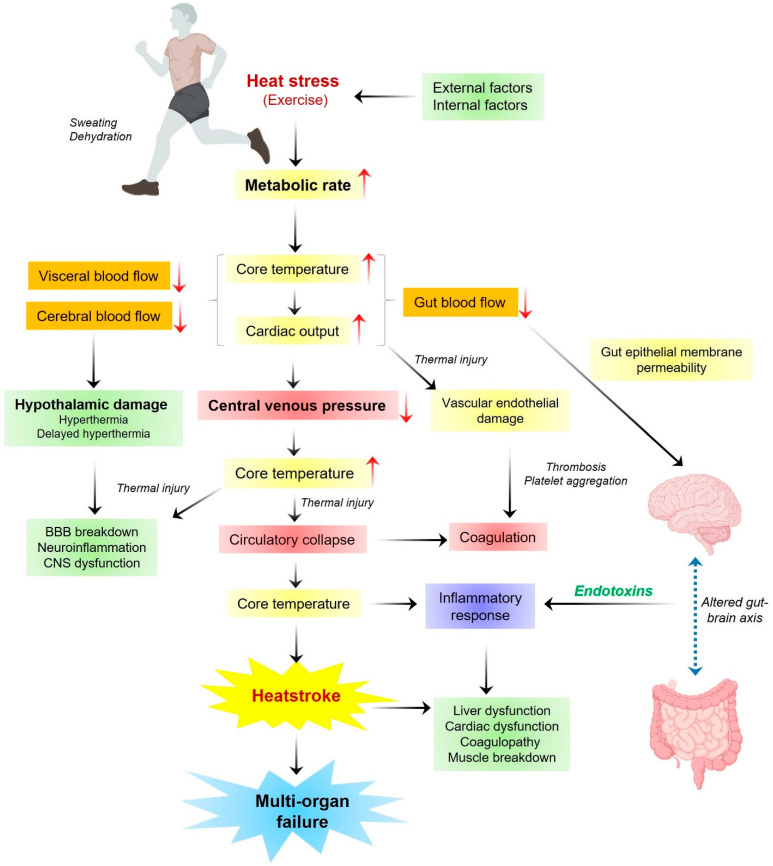
A general overview of sequential events during heatstroke (top to bottom). The red upright and downward arrows show increases and decreases in the body functions, respectively. The dotted double-headed blue arrow indicates a dynamic axis between the gut and brain during heatstroke.

**Figure 3:**
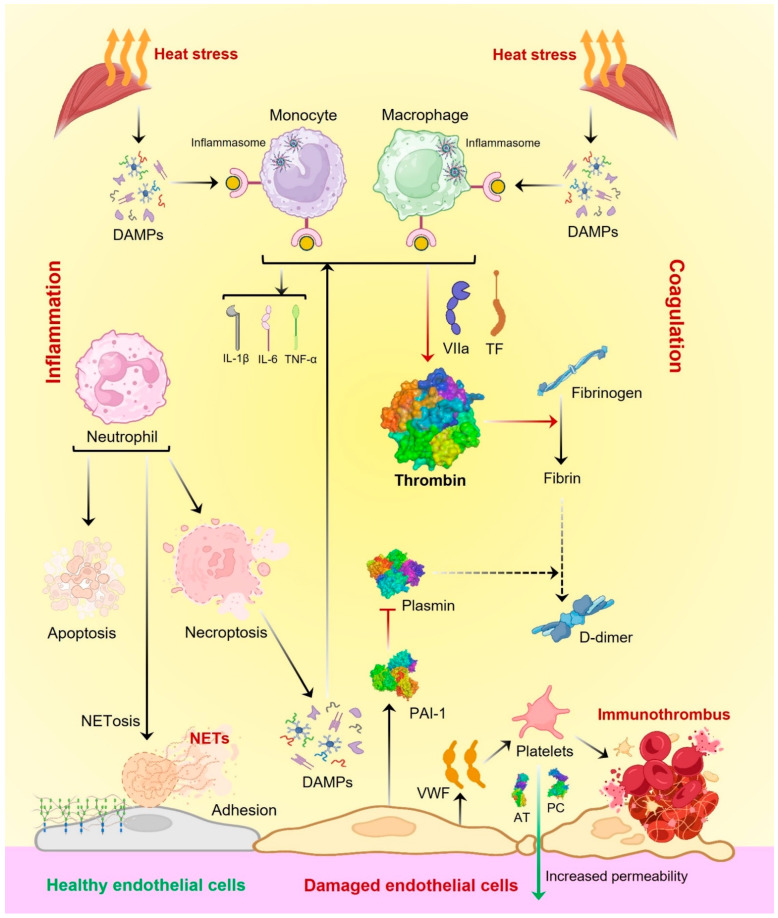
Molecular pathogenesis of heatstroke. The black arrows represent the sequential processes. The red arrows show inhibition. The blunt end red arrow shows complete blocking. The dotted black arrow indicates a slowdown of the normal process while the green arrow shows a rapid increase. DAMPs: damage-associated molecular patterns, TF: tissue factor, VIIa: factor VIIa, PAI-1: plasminogen activator inhibitor 1, VWF: Von Willebrand factor, AT: antithrombin, and PC: protein C.

Heatstroke primarily occurs when cardiac output is inadequate to meet the high thermoregulatory demands of the body, causing a transition from a compensable thermoregulatory phase in which heat loss exceeds heat gain to a noncompensable phase in which heat gain exceeds heat loss. This imbalanced thermoregulatory process ultimately results in a persistent increase in core body temperature, which sets off a malicious cycle of events that eventually results in multiorgan dysfunction or failure via directly inducing cytotoxicity, inflammatory response, and coagulation [13,14] (Figure 2 and Figure 3). Conclusively, heatstroke is a detailed phenomenon where various factors may be involved depending on the environmental or individual physiological conditions. The present review is mainly focused on the inflammatory responses and associated molecular biomarkers during heatstroke.

### 3.1. Inflammatory Immune Response in Heatstroke

The series of inflammatory response events generated during heatstroke is not fully understood yet and needs to be further studied. Importantly, a coordinated stress response triggered via hyperthermia during heatstroke involving endothelial cells, leukocytes, and epithelial cells plays a role in cell repair and protection against tissue damage. This response is regulated by the heat-shock protein family and ultimately results in altered levels of pro-inflammatory and anti-inflammatory cytokines in plasma and tissues [15,16]. In the case of long-term hyperthermia, this inflammatory response becomes uncontrolled and results in acute physiological conditions such as hypoxia, circulatory failure, and higher metabolic demands along with direct heat-associated cytotoxicity [17,18]. Next, the heatstroke-associated inflammatory response is equivalent to the systemic inflammatory response syndrome (SIRS). Interestingly, SIRS is regulated by circulating messenger RNAs that stimulate the release of cytokines and high-mobility group box 1 protein (HMGB1), which in turn causes the overactivation of leukocytes (monocytes, and macrophages) and endothelial cells. Further, cytokines play an important role in heatstroke-associated inflammatory response, which is also connected with SIRS. Notably, an increased circulatory concentration of cytokines including IL-1α, IL-1β, soluble IL-6 receptor (sIL-6R), IL-6, IL-8, IL-10, IL-12, IFN-γ, tumor necrosis factor (TNF-α), and sTNFR has been reported during the heatstroke and becomes normal shortly after cooling [19]. Next, inflammatory cytokines activate neutrophils to stimulate apoptosis, necroptosis, and NETosis. As a result, heat-induced tissue injury generates damage-associated molecular patterns (DAMPs), along with neutrophil extracellular traps (NETs) that stimulate immune cells to produce inflammatory cytokines and vice versa via inflammasomes. Furthermore, ruptured adhesive neutrophil damage and NETs simultaneously damage the endothelial cells and induce thrombosis. Concurrently, activated immune cells expressed tissue factors (TFs) and factor VIIa that induce coagulation. Coagulation torrent generates thrombin, a demanding mediator of disseminated intravascular coagulation (DIC) that is sequentially involved in inflammation progression and coagulation which finally results in endothelial cell damage via the formation of immunothrombus (Figure 3). Further, endothelial cell damage reduces fibrinolysis by activated plasminogen activator inhibitor 1 (PAI-1) generation and enhanced permeability that results in reduced availability of natural anticoagulants such as antithrombin (AT) and protein C (PC). At the same time, damaged endothelial cells secrete von Willebrand factor (VWF) that induces platelet aggregation and finally the formation of an inflammatory thrombus (Figure 3). Overall, SIRS with other associated immune modulation during heatstroke causes DIC, leading to multiple organ failure, and even death (Figure 3) [1,20]. Additionally, the gut–brain barrier becomes permeable during heatstroke, allowing the migration of bacterial endotoxins and lipopolysaccharides to the circulatory system and ultimately to the brain, which causes neuroinflammation [21]. In conclusion, endotoxemia and enhanced LPS levels are suggested to be involved in the induction of inflammatory response during heatstroke [22]. Disruption of the gut–brain axis and increased intestinal permeability have already been observed in exertional hyperthermia; however, the detailed mechanism is not well understood [23,24]. Overall, the function of cytokines in SIRS, DIC, and the role of gut–brain axis dysbiosis in heatstroke need to be further explored.

### 3.2. Neuroinflammation Triggered by Heatstroke

Other than the common inflammatory response, neuroinflammation is one of the main characteristics of heatstroke that leads to neurological impairment. Heatstroke-associated neurological impairment might appear in different forms such as milder symptoms including behavioral abnormalities, disorientation, fatigue, delirium, or weakness while severe symptoms include seizures and altered consciousness levels [25]. There are several theories on the causes of hyperthermic cerebral dysfunction. As suggested by one such theory, at temperatures higher than 38–39 °C, the blood–brain barrier becomes more permeable, which leaves the brain vulnerable to intestinal and systemic toxins like LPS37. Additionally, reduced cerebral oxygen levels and glucose consumption cause restricted metabolic activities in the brain or impaired cellular absorption, resulting in brain injury [26].

Common inflammatory response leads to neuroinflammation and profound dysfunction of the CNS during heatstroke. Increased levels of IL-1β have been reported during heatstroke [19]. In a recent study, an inflammatory response is induced using lipopolysaccharide (LPS) or lipoteichoic acid (LTA) injection in NLRP3 knockdown mice. The results suggested improved heat tolerance and a reduction in heatstroke-induced death via diminishing IL-1β production in mice hypothalamus. Also, administration of IL-1β neutralizing antibodies confirmed an increased survival under heatstroke conditions [27]. In conclusion, inflammatory cytokines produced during heatstroke play an essential role in the development of neuroinflammation and lead to death. Further, mesenchymal stem cells (MSCs) are suggested to have neuroprotective roles in heatstroke-induced neuroinflammation and CNS injury. It has been shown in high-temperature-induced heatstroke rat models that tail vein delivery of MSCs was able to reduce the inflammatory response and mortality efficiently. Also, MSC administration was found to restore heatstroke-induced hippocampal damage, and astrocytes along with efficient inhibition of cerebral inflammatory response [28]. These results suggest a potential role of MSC therapy in alleviating heatstroke-induced neuroinflammation. Additionally, apoptosis in heatstroke is illuminated in recent studies by JAK2 (Janus kinase 2), which is a signal transducer and activator of the transcription (STAT) pathway. This pathway is vital for angiogenesis and of significance in the hepar and myocardium, which are the most vulnerable organs during heat stress. Both JAK2 and STAT3 seem to have a protective mechanism during heat stress as they lessen the impact of ischemic injury by inhibiting the apoptotic processes. A recent study demonstrated the role of JAK2 in heatstroke-associated brain injury and inflammatory response by employing AG490, a JAK2 inhibitor. Heatstroke-induced rats showed edema in hippocampal tissues, increased apoptotic rate, upregulation of malondialdehyde (MDA), nitric oxide synthase (iNOS), reactive oxygen species (ROS), along with downregulation of superoxide dismutase (SOD) when compared to the control group. Enhanced secretion and upregulation of other heatstroke-induced inflammatory factors including matrix metallopeptidase 2 and 9 (MMP2 and MMP-n 9), intercellular adhesion molecule-1 (ICAM-1), tumor necrosis factor-beta1 (TNF-β1) and cyclooxygenase-2 (COX-2) were also observed. In conclusion, the study suggested an important role of JAK2/STAT3 pathways in heatstroke-associated neuroinflammation [29]. Although available experimental evidence suggests neuroinflammation as one of the major pathologies during heatstroke, further detailed studies are required to understand and develop treatment strategies to overcome the associated mortalities.

## 4. Clinical Overview of Heatstroke and Associated Dysfunctions

Although a thorough evaluation of the clinical picture of heatstroke has already been performed, updates are needed in the current phase of rapid changes in environmental stress and human lifestyle [12,30]. Heatstroke is categorized as classic or exertional heatstroke, which includes three phases, named as the hyperthermic (neurologic acute phase), the hematologic–enzymatic phase (24–48 h after the event), and the late renal–hepatic phase (only if clinical symptoms remain for 96 h). Importantly, the acute neurologic phase is most crucial to detecting and treating and the accurate core body temperature plays an important role in defining the heatstroke-associated sickness; however, hyperthermia during excessive physical efforts does not necessarily represent heatstroke [31]. Specifically, heatstroke patients are characterized by abnormalities with the CNS due to the high sensitivity of the brain toward heat-associated fatigue. In serious cases, exertional heatstroke may result in brain edema followed by seizures and sphincter incontinence along with some early signs of illness including behavioral changes, disorientation, delirium, vertigo, weakness, agitation, aggression, slurred speech, nausea, and vomiting [2,17]. Additionally, there is the possibility of long-term neural damage to the autonomic and enteric nervous systems during heatstroke. Also, in severe exertional heatstroke, multiorgan dysfunction may occur within the initial 24 to 48 h. In most cases, clinical indications of heatstroke are reduced if immediate therapeutics are applied and most patients recover without any long-term consequences. However, acute renal, cardiac, and hepatic dysfunction and failure, acute respiratory distress syndrome, and prolonged altered consciousness are among the potential side effects of exertional heatstroke [2,32]. Next, cerebellar ataxia, dysarthria, cognitive problems, and anterograde amnesia are a few examples of neurologic sequelae that might last for weeks or months, following exertional heatstroke [33]. In addition, autopsy investigations following exertional heatstroke also suggest extensive microthrombosis, bleeding, and inflammatory damage along with heat-induced necrosis and apoptotic cell death, which is the main cause of final end-organ failure (Figure 2) [18,30,34]. Overall, heatstroke is a life-threatening clinical condition that might lead to death if the right measures are not taken within the right time frame.

## 5. Heat-Associated Protein Misfolding and Aggregation

Temperature can trigger protein misfolding by disrupting the delicate balance of interactions that maintain a protein’s native, functional structure. Proteins are held together in their folded state by various non-covalent forces, such as hydrogen bonds, hydrophobic interactions, van der Waals forces, and electrostatic interactions. When the temperature increases, these interactions can weaken, leading to protein destabilization and misfolding. Elevated temperatures raise the kinetic energy of molecules, including proteins, which in turn accelerates their movement and interactions. The connection between temperature and protein kinetics can be understood through the principles of thermodynamics and reaction rate theory. Proteins undergo various conformational changes as part of their function as both catalytic and substrate. These changes often involve transitions between different energetic states. Higher temperatures speed up these conformational transitions, allowing proteins to fold/unfold or switch between active and inactive forms more rapidly. This faster motion often leads to increased catalytic turnover. The collision theory states that chemical reactions occur when reactant molecules collide with sufficient energy and proper orientation. At higher temperatures, proteins and substrate molecules move faster, which leads to an increase in the number of effective collisions per unit of time. Higher temperatures can also enhance protein-protein interaction kinetics by increasing the frequency and energy of collisions between proteins.

**Figure 4:**
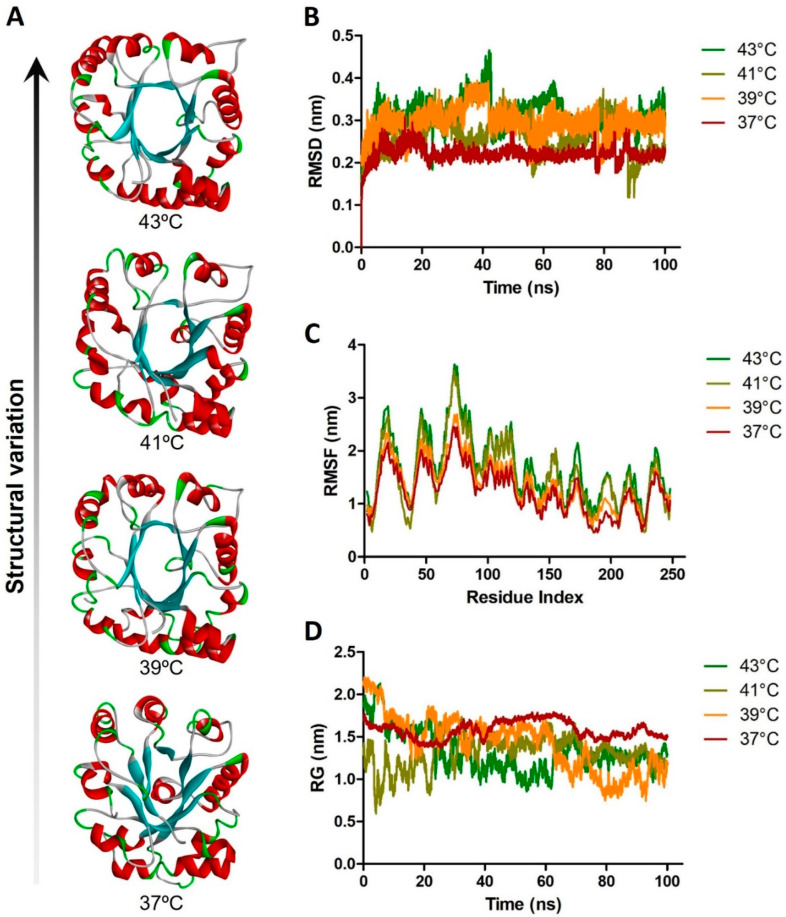
Molecular dynamics simulation analysis of trios phosphate isomerase (PDB: 8TIM) at different temperatures to demonstrate the structural variations. (**A**) The solid ribbon structures of trios phosphate isomerase at 37 °C, 39 °C, 41 °C, and 43 °C from bottom to top, respectively. (**B**) RMSD, (**C**) RMSF, and (**D**) RG values of trios phosphate isomerase during a 100 ns molecular dynamics and simulation experiment at 37 °C, 39 °C, 41 °C, and 43 °C, respectively. The maroon line shows the values at 37 °C which are consistent in comparison to at the higher temperatures.

Therefore, increasing the temperature enhances protein kinetics by raising the kinetic energy of molecules, leading to faster movements and more collisions. Increasing the rate of biochemical reactions by enabling more molecules to overcome activation energy barriers and speeding up conformational changes, turnover rates, and diffusion all contribute to faster biochemical processes. However, excessive temperature increases can lead to denaturation, where proteins lose their native structure and function, misfolded, and more frequent collisions of misfolded proteins increase the likelihood of aggregation. To demonstrate this phenomenon, a molecular dynamics and simulation experiment was performed by employing a glycolytic housekeeping enzyme, triose phosphate isomerase (PDB: 8TIM). Our analysis clearly showed the increased structural fluctuation with elevated temperatures in terms of RMSF (root mean square fluctuation), RMSD (root mean square deviation), and RG (radiation of gyration) (Figure 4). The results demonstrate how a little variation in ambient temperature might alter the functional structure of housekeeping enzymes during heatstroke and result in a life-threatening condition leading to death.

## 6. Heatstroke Biomarkers

Experimental data indicate that heatstroke-induced inflammatory response and other associated symptoms vary according to the different body states and are affected by multiple other factors in a tissue- and time-specific manner. Also, many times, available measures may not properly represent the severity of the illness or long-term effects of heatstroke. Based on available studies, some biomarkers can be considered to estimate disease severity and accurate diagnosis; however, further in vivo screening and standardization in different clinical conditions is required. HMGB1 is reported as a severity indication and an important mortality predictor in exertional heatstroke. Peak levels of HMGB1 were observed within 6 to 13 h post-heatstroke and confirmed to have been present in circulation at an early stage. Interestingly, plasma levels of HMGB1 significantly remained enhanced in the subsequent 6 days post-heatstroke when compared to healthy individuals [35]. Next, heatstroke is also associated with acute kidney injuries. In a canine heatstroke study, acute kidney injuries are characterized using different biomarkers including urine neutrophil gelatinase-associated lipocalin (UNGAL), urine retinol-binding protein (URBP), and urine C-reactive protein (UCRP). Increased levels of all these biomarkers were reported during heatstroke; however, extremely high levels of UNGAL were detected [36]. Another study reported elevated levels of troponin I as a potential marker of myocardial damage following heatstroke. Additionally, lower levels of urinary heat-shock protein 72 were reported to be associated with acute kidney injuries in dogs, which further suggests a link between heatstroke and acute kidney injuries [37]. Exertional heatstroke is also reported to be associated with malignant hyperthermia in studies involving well-trained and adapted patients, even when there were no extreme environmental conditions or physical activity [38,39]. Similarly, exertional heatstroke was found to be associated with malignant hyperthermia in a recent case report of a highly trained athlete. Interestingly, the athlete was known to have a malignant hyperthermia-associated mutation in the RyR1 gene, which suggests a link between exertional heatstroke and malignant hyperthermia; however, further detailed studies are required in the respective field [40].

Heatstroke-induced inflammatory response is well reported, but not understood. Recent studies showed that histone leaking from damaged cells in the extracellular environment plays an important role in pathologies characterized as toxic, pro-inflammatory, and pro-thrombotic. Interestingly, elevated levels of serum histones are detected during heatstroke in canines and are also associated with disease severity such as oxidation, inflammation, and coagulation [41]. Heatstroke is also known to be associated with intestinal injuries that further induce bacterial translocation and endotoxemia; however, the full mechanism needs to be understood. It has been observed that cyptdin-2, an intestinal α-defensin, is upregulated during heatstroke in mice intestinal tissues as the administration of ulinastatin, a multivalent enzyme inhibitor that downregulates cyptdin-2 expression, was found to alleviate heatstroke pathologies [42]. Overall, results obtained from the recent studies showed the potential of HMGB1, UNGAL, URBP, UCRP, urinary heat-shock protein 72, cardiac troponin I, serum histones, and cyptdin-2 as biomarkers or novel predictors for the early onset of heatstroke. However, heatstroke still needs to be defined well in different environmental and clinical conditions, including individual differences, to be further tested or approved for clinical therapeutic uses.

## 7. Therapeutic Strategies for the Treatment of Heatstroke-Induced Injuries

Heatstroke pathogenesis is a sequential process that primarily includes an inflammatory response followed by oxidative stress, coagulation, and finally multiorgan failure. Similarly, heatstroke treatment strategies are also categorized into different phases. Primary treatment includes heat dissipation strategies and electrolyte replacement along with cooling therapies. Primary treatment is followed by pharmacotherapy using clinical drugs according to the need (Table 1 and Table 2). Targeted temperature management is one of the important strategies in the early stage of heatstroke including fluid resuscitation, and blood purification [43,44]. Enhanced internal and external cooling and improved ventilation strategies along with cold saline intravenous drips have been clinically recommended to decrease the patient’s body temperature during heatstroke [45]. Total cold water immersion therapy including ice water-soaked towels and ice packs has been clinically proven to reduce heatstroke-induced core body temperature [46,47]. Next, hypothermic retrograde jugular vein flush is suggested for brain cooling by reducing systemic inflammation, oxidative stress, ischemic injury, and coagulation during heatstroke [48,49]. Additionally, traditional Chinese medicines Huoxiang Zhengqi liquid and Angong Niuhuang Wan were reported to have beneficial effects in alleviating heatstroke symptoms including body heat, acute intestinal injury, and cerebral hemorrhage [50,51]. Further, clinical drugs such as dexamethasone, methylprednisolone, hydrocortisone, and epinephrine have been frequently used to lessen the heatstroke-induced inflammatory response [44,52]. Propofol is an FDA-approved anesthetic known to diminish heatstroke-induced cellular damage in preclinical studies via inhibition of intracellular HMGB1 release [53]. Next, ibuprofen, a nonsteroidal painkiller drug, showed protection against intestinal damage during exertional heatstroke [54].

Further, heatstroke-induced injuries are often associated with oxidative stress so antioxidant therapies are reported and considered as one of the promising treatment strategies to alleviate heatstroke-associated injuries. Chinese herbal medicines such as Magnolol and flavonoid baicalin were found to be beneficial in reducing cerebral ischemia and oxidative stress during heatstroke. Additionally, it has been observed that regular exercise for a minimum of 3 weeks results in a reduction in heatstroke-induced ROS and lipid peroxidation [58]. Additionally, there are several other potential measures in preclinical studies to mitigate heatstroke-induced injuries that include cellular therapies, cytokine antagonists, bone marrow-derived mononuclear cell transplantation therapy, and human umbilical cord blood-derived CD34+ cell therapy (Table 2). Even after showing the potential results in preclinical studies, these therapies have to go under clinical trials before their actual applications in human therapeutics.

## 8. Discussion and Future Perspectives

Although the criteria for exertional heatstroke casualty have been described in detail, there are still many loops and holes. Athletes, employees, recreational exercisers, and members of the armed forces are among the groups in most danger from the growing threat of increased heat stress brought on by climate change. The most important point is the identification of heatstroke-related symptoms before the actual onset of illness. Also, there is disagreement over the critical temperature ranges at which active cooling should cease once cooling measures have been implemented. It is necessary to conduct further research to determine the effects of cooling on the cardiovascular system, hemodynamics, and associated morbidity and mortality. Further, there are insufficient biomarkers to forecast the severity of heatstroke or other associated dysfunctions. Interestingly, genetic factors might also play an important role in heatstroke outcomes such as susceptibility to temperature changes and heat injury. A recent gene expression profiling study of humans under exertional heatstroke revealed a significant downregulation of interleukins and stress-associated genes that might be involved in heatstroke injuries [81]. Next, specific mutations such as p.K1393R in the ryanodine receptor (RyR1) gene, a typical malignant hyperthermia mutation, were found to be associated with altered muscle oxidative metabolism and involved in heatstroke-related rhabdomyolysis [40]. Another hyperthermia-associated mutation, Y522S, in the RyR1 gene, causes Ca^2+^ leakage and is responsible for the generation of reactive nitrogen species and subsequent S-nitrosylation of the RyR1 gene. Overall, the mutant RyR1 gene results in increased temperature sensitivity that is involved in muscle spasms, followed by heatstroke and death in mice [82]. Notably, there is Cpd1 (6,7-(methylenedioxy)-1-octyl-4-quinolone-3-carboxylic acid), a selective RyR1 inhibitor that can protect against malignant hypothermia and heatstroke injuries by restricting Ca^2+^ leakage [83]. Further, a calcium-binging protein, Calsequestrin-1 was found to be associated with malignant hypothermia and heatstroke; however, further studies are required to confirm its clinical relevance [84]. Moreover, heat tolerance-associated genetic defects might result in a higher risk and susceptibility for heatstroke, so it is important to study heat tolerance-related genetics. Also, adaptation to environmental conditions plays an important role and many generations in the same type of climatic conditions showed an adaptation to high temperatures as a heritable characteristic [44]. Considering the facts, acquired standardized heat adaptation time-bound training is a must for the specific group of people at high risk of heatstroke including military personnel, athletes, and specialized workers.

In conclusion, further detailed studies are required to understand the complete genetic variations involved in heatstroke injuries. These genetic traits may also develop as potential biomarkers for early diagnosis of susceptibility to heat injury. Additionally, tissue- or molecular-level characterization of heatstroke-associated illness or dysfunctions is required. Especially, the increased permeability of the gut and the role of the gut–brain axis in heatstroke are not yet fully understood. Interestingly, gut-dysbiosis and identification of specifically involved gut microbiota might serve as novel biomarkers for heatstroke. Also, detailed in vivo validation of biomarkers is required in different time point-specific conditions. Additionally, there are no outcomes data to support the return-to-play decision-making procedure, which should be established based on best practices. Overall, these gaps need to be filled for the development of better therapeutics for heatstroke treatment.

## Figures and Tables

**Table 1:** Selected clinical or preclinical therapies along with mechanisms of action to mitigate heatstroke-induced injuries.

Treatment/Therapy	Mechanism of Action	References
Targeted temperature management	Maintains body temperature.	[43,44]
Total cold water immersion	Maintains body temperature.	[46,47]
Hypothermic retrograde jugular vein flush	Reduces systemic inflammation, oxidative stress, ischemic injury, and coagulation.	[48,49]
Bone marrow-derived mononuclear cell (BMMNC) transplantation therapy	Reduces acute systemic inflammation and vascular endothelial injury by reducing proinflammatory cytokines.	[55]
Human umbilical cord blood-derived CD34+ cell therapy	Protects against multiorgan dysfunction by reducing systemic inflammation.	[56]
Mesenchymal stem cell therapy	Reduces mortality by protecting against neurological defects and hippocampal damage, by restricting the overactivation of hippocampal microglia.	[28,57]
Regular physical exercise for at least 3 weeks	Reduction in heatstroke-induced ROS and lipid peroxidation.	[58]

**Table 2:** Suggested preclinical potential drugs and strategies for the treatment of heatstroke-induced inflammatory response and associated injuries.

Drugs/Strategies	Source	Action Against Heatstroke Pathologies	Study Model	References
Dexmedetomidine	Synthetic	Reduces neuroinflammation by activating PI3K/Akt via TREM2 in microglia.	Mice	[59]
Eicosapentaenoic acid	Cold water fish	Maintaining intestinal barrier permeability by protecting tight junctions and reducing plasma endotoxin levels.	Rats	[60]
Alad-1	Synthetic	An agonist of aldehyde dehydrogenase 2, alleviates oxidative stress by reducing ROS.	Mice	[61]
17-dimethylaminoethylamino-17-demethoxy-geldanamycin (17-DMAG)	Semisynthetic derivative of geldanamycin	Decreases hypotension and organ dysfunction via upregulation of Hsp70 and phosphorylation of AMPK.	Rats	[62]
Misoprostol	Synthetic	Prostaglandin protects against multiorgan dysfunction and intestinal damage.	Rats	[63]
l-Arginine	Natural amino acid	Protects intestinal epithelial integrity by replenishing E-calmodulin downregulation.	Human colorectal adenocarcinoma (Caco-2) cells	[64]
Melatonin	Natural hormone	Diminishes hyperthermia and hypotension, and protects against myocardial injury by attenuating oxidative stress and inflammation. Reduces neutrophil infiltration and gene expression of pro-inflammatory factors.	Rats	[65,66,67]
Salidroside	Roots of *Rhodiola rosea*	Protects against myocardial injury by reducing inflammation and oxidative stress.	Mice	[68]
Quercetin	Plant pigment (flavonoid)	Protects against myocardial injury by reducing inflammation and oxidative stress.	Rats	[69]
Geranylgeranylacetone	Synthetic	Protects against hepatic and renal dysfunction by inducing HSP70, and anti-inflammatory cytokines.	Rats	[70]
Allopurinol	Synthetic	Reduces portal vein endotoxins and protects the integrity of the circulatory and intestinal barrier.	Rats	[71]
Sodium tanshinone IIA	Derivative of tanshinone IIA (extracted from *Salvia miltiorrhiza*)	Protects against multiorgan failure by reducing inflammatory response, intravascular coagulation, and endothelial cell apoptosis.	Rats	[72]
Magnolol	Bark of Magnolia tree	Reduces oxidative damage.	Rats	[73]
Baicalin	Roots of *Scutellaria baicalensis*	Protects against cerebrovascular dysfunction and reduces brain inflammation.	Rats	[58]
PARP inhibitor	Synthetic	Reduces heatstroke injury via inducing the expression of HSPs.	Mice	[74]
IL-1 receptor antagonist	Naturally produced by the body	Protects neuronal damage by restricting cerebral ischemia.	Rats	[75]
HMGB1 monoclonal antibody	Synthetic	Alleviates inflammatory response and reduces liver injury.	Rats	[76]
Recombinant human thrombomodulin	Synthetic	Protects against disseminated intravascular coagulation.	Humans	[77]
Mannitol	Natural sugar alcohol	Reduces intestinal damage via inhibition of RIPK1/RIPK3-dependent necroptosis.	Mice	[78]
α-tocopherol	Natural	Reduces intestinal damage via inhibition of RIPK1/RIPK3-dependent necroptosis.	Mice	[78]
N-acetyl-l-cysteine	Synthetic	Reduces intestinal damage via inhibition of RIPK1/RIPK3-dependent necroptosis.	Mice	[78]
Huanglian	Extracted from the rhizome of *Coptis chinensis*	Protects against brain injury via attenuating hypothermia and neuroinflammation and upregulation of HSPs and c-Fos.	Mice	[79]
*Bacillus licheniformis*	Bacteria	Reduces intestinal damage by maintaining the integrity of the intestinal barrier and improving the health of gut microbiota.	Rats	[80]

## Data Availability

No new data were created or analyzed in this study. Data sharing is not applicable to this article.

## References

[1] Hifumi T., Kondo Y., Shimizu K., Miyake Y. (2018). Heat stroke. J. Intensive Care.

[2] Shapiro Y., Seidman D.S. (1990). Field and clinical observations of exertional heat stroke patients. Med. Sci. Sports Exerc..

[3] Ballester J., Quijal-Zamorano M., Méndez Turrubiates R.F., Pegenaute F., Herrmann F.R., Robine J.M., Basagaña X., Tonne C., Antó J.M., Achebak H. (2023). Heat-related mortality in Europe during the summer of 2022. Nat. Med..

[4] Hajat S., Vardoulakis S., Heaviside C., Eggen B. (2014). Climate change effects on human health: Projections of temperature-related mortality for the UK during the 2020s, 2050s and 2080s. J. Epidemiol. Community Health.

[5] Misset B., De Jonghe B., Bastuji-Garin S., Gattolliat O., Boughrara E., Annane D., Hausfater P., Garrouste-Orgeas M., Carlet J. (2006). Mortality of patients with heatstroke admitted to intensive care units during the 2003 heat wave in France: A national multiple-center risk-factor study. Crit. Care Med..

[6] Mahant S. (2015). The evaluation and management of heat injuries in an intensive care unit. Indian J. Crit. Care Med..

[7] Bouchama A., Knochel J.P. (2024). Heat Stroke. N. Engl. J. Med..

[8] Kravchenko J., Abernethy A.P., Fawzy M., Lyerly H.K. (2013). Minimization of heatwave morbidity and mortality. Am. J. Prev. Med..

[9] Bouchama A., Dehbi M., Mohamed G., Matthies F., Shoukri M., Menne B. (2012). Prognostic Factors in Heat Wave—Related Deaths. Arch. Intern. Med..

[10] Rowland T. (2008). Thermoregulation during exercise in the heat in children: Old concepts revisited. J. Appl. Physiol..

[11] Robertson B., Walter E. (2010). “Cool runnings”: Heat stroke in cool conditions. Emerg. Med. J..

[12] Bouchama A., Abuyassin B., Lehe C., Laitano O., Jay O., O’Connor F.G., Leon L.R. (2022). Classic and exertional heatstroke. Nat. Rev. Dis. Prim..

[13] Garcia C.K., Renteria L.I., Leite-Santos G., Leon L.R., Laitano O. (2022). Exertional heat stroke: Pathophysiology and risk factors. BMJ Med..

[14] Lim C.L. (2018). Heat sepsis precedes heat toxicity in the pathophysiology of heat stroke—A new paradigm on an ancient disease. Antioxidants.

[15] Yan Y.E., Zhao Y.Q., Wang H., Fan M. (2006). Pathophysiological factors underlying heatstroke. Med. Hypotheses.

[16] Dehbi M., Baturcam E., Eldali A., Ahmed M., Kwaasi A., Chishti M.A., Bouchama A. (2010). Hsp-72, a candidate prognostic indicator of heatstroke. Cell Stress Chaperones.

[17] Leon L.R., Bouchama A. (2015). Heat stroke. Compr. Physiol..

[18] Bouchama A., Roberts G., Al Mohanna F., El-Sayed R., Lach B., Chollet-Martin S., Ollivier V., Al Baradei R., Loualich A., Nakeeb S. (2005). Inflammatory, hemostatic, and clinical changes in a baboon experimental model for heatstroke. J. Appl. Physiol..

[19] Leon L.R., Helwig B.G. (2010). Heat stroke: Role of the systemic inflammatory response. J. Appl. Physiol..

[20] Caserta S., Mengozzi M., Kern F., Newbury S.F., Ghezzi P., Llewelyn M.J. (2018). Severity of Systemic Inflammatory Response Syndrome Affects the Blood Levels of Circulating Inflammatory-Relevant MicroRNAs. Front. Immunol..

[21] Liu X., Li H., Lu A., Zhong Y., Hou X., Wang N., Jia D., Zan J., Zhao H., Xu J. (2012). Reduction of intestinal mucosal immune function in heat-stressed rats and bacterial translocation. Int. J. Hyperth..

[22] Heled Y., Fleischmann C., Epstein Y. (2013). Cytokines and their role in hyperthermia and heat stroke. J. Basic Clin. Physiol. Pharmacol..

[23] Walter E., Gibson O.R., Stacey M., Hill N., Parsons I.T., Woods D. (2021). Changes in gastrointestinal cell integrity after marathon running and exercise-associated collapse. Eur. J. Appl. Physiol..

[24] Walter E., W Watt P., Gibson O.R., Wilmott A.G.B., Mitchell D., Moreton R., Maxwell N.S. (2021). Exercise hyperthermia induces greater changes in gastrointestinal permeability than equivalent passive hyperthermia. Physiol. Rep..

[25] Walter E.J., Carraretto M. (2016). The neurological and cognitive consequences of hyperthermia. Crit. Care.

[26] Cremer O.L., Kalkman C.J. (2007). Cerebral pathophysiology and clinical neurology of hyperthermia in humans. Prog. Brain Res..

[27] Zhang Z.T., Gu X.L., Zhao X., He X., Shi H.W., Zhang K., Zhang Y.M., Su Y.N., Zhu J.B., Li Z.W. (2021). NLRP3 ablation enhances tolerance in heat stroke pathology by inhibiting IL-1β-mediated neuroinflammation. J. Neuroinflamm..

[28] Zhang Y., Deng Z., Li Y., Yuan R., Yang M., Zhao Y., Wang L., Zhou F., Kang H. (2020). Mesenchymal Stem Cells Provide Neuroprotection by Regulating Heat Stroke-Induced Brain Inflammation. Front. Neurol..

[29] Tao Z., Cheng M., Wang S.C., Lv W., Hu H.Q., Li C.F., Cao B.Z. (2015). JAK2/STAT3 pathway mediating inflammatory responses in heatstroke-induced rats. Int. J. Clin. Exp. Pathol..

[30] Malamud N., Haymaker W., Custer R.P. (1946). Heat stroke; a clinico-pathologic study of 125 fatal cases. Mil. Surg..

[31] Maron M.B., Wagner J.A., Horvath S.M. (1977). Thermoregulatory responses during competitive marathon running. J. Appl. Physiol. Respir. Environ. Exerc. Physiol..

[32] Wang C.C., Tsai M.K., Chen I.H., Hsueh C.W., Shiang J.C. (2008). Heat stroke. J. Intern. Med. Taiwan.

[33] Yang M., Li Z., Zhao Y., Zhou F., Zhang Y., Gao J., Yin T., Hu X., Mao Z., Xiao J. (2017). Outcome and risk factors associated with extent of central nervous system injury due to exertional heat stroke. Medicine.

[34] Roberts G.T., Ghebeh H., Chishti M.A., Al-Mohaiina F., El-Sayed R., Al-Mohanna F., Bouchama A. (2008). Microvascular injury, thrombosis, inflammation, and apoptosis in the pathogenesis of heatstroke a study in baboon model. Arterioscler. Thromb. Vasc. Biol..

[35] Tong H.S., Tang Y.Q., Chen Y., Qiu J.M., Wen Q., Su L. (2011). Early elevated HMGB1 level predicting the outcome in exertional heatstroke. J. Trauma Inj. Infect. Crit. Care.

[36] Segev G., Daminet S., Meyer E., De Loor J., Cohen A., Aroch I., Bruchim Y. (2015). Characterization of kidney damage using several renal biomarkers in dogs with naturally occurring heatstroke. Vet. J..

[37] Bruchim Y., Avital Y., Horowitz M., Mazaki-Tovi M., Aroch I., Segev G. (2017). Urinary heat shock protein 72 as a biomarker of acute kidney injury in dogs. Vet. J..

[38] Capacchione J.F., Muldoon S.M. (2009). The relationship between exertional heat illness, exertional rhabdomyolysis, and malignant hyperthermia. Anesth. Analg..

[39] Grogan H., Hopkins P.M. (2002). Heat stroke: Implications for critical care and anaesthesia. Br. J. Anaesth..

[40] Poussel M., Guerci P., Kaminsky P., Heymonet M., Roux-Buisson N., Faure J., Fronzaroli E., Chenuel B. (2015). Exertional heat stroke and susceptibility to malignant hyperthermia in an athlete: Evidence for a link?. J. Athl. Train..

[41] Bruchim Y., Ginsburg I., Segev G., Mreisat A., Avital Y., Aroch I., Horowitz M. (2017). Serum histones as biomarkers of the severity of heatstroke in dogs. Cell Stress Chaperones.

[42] Ji J., Gu Z., Li H., Su L., Liu Z. (2018). Cryptdin-2 predicts intestinal injury during heatstroke in mice. Int. J. Mol. Med..

[43] Stanger D., Mihajlovic V., Singer J., Desai S., El-Sayegh R., Wong G.C. (2018). Editor’s Choice-Effects of targeted temperature management on mortality and neurological outcome: A systematic review and meta-analysis. Eur. Hear. J. Acute Cardiovasc. Care.

[44] Liu S.Y., Song J.C., Mao H.D., Zhao J.B., Song Q., Liu D.W., Yu K.J., Li J.G., Lin H.Y., Lin S. (2020). Expert consensus on the diagnosis and treatment of heat stroke in China. Mil. Med. Res..

[45] Bouchama A., Dehbi M., Chaves-Carballo E. (2007). Cooling and hemodynamic management in heatstroke: Practical recommendations. Crit. Care.

[46] Armstrong L.E., Crago A.E., Adams R., Roberts W.O., Maresh C.M. (1996). Whole-body cooling of hyperthermic runners: Comparison of two field therapies. Am. J. Emerg. Med..

[47] Douma M.J., Aves T., Allan K.S., Bendall J.C., Berry D.C., Chang W.T., Epstein J., Hood N., Singletary E.M., Zideman D. (2020). First aid cooling techniques for heat stroke and exertional hyperthermia: A systematic review and meta-analysis. Resuscitation.

[48] Hsu S.F., Niu K.C., Lin C.L., Lin M.T. (2006). Brain cooling causes attenuation of cerebral oxidative stress, systemic inflammation, activated coagulation, and tissue ischemia/injury during heatstroke. Shock.

[49] Wen Y.S., Huang M.S., Lin M.T., Lee C.H. (2004). Hypothermic retrograde jugular vein flush in heatstroke rats provides brain protection by maintaining cerebral blood flow but not by hemodilution. Crit. Care Med..

[50] Li Y.N., Tao H., Hong J.H., Xiong Y.L., Pan X.C., Liu Y., Yang X.S., Zhang H.G. (2022). The Chinese Herbal Formula Huoxiang Zhengqi Dropping Pills Prevents Acute Intestinal Injury Induced by Heatstroke by Increasing the Expression of Claudin-3 in Rats. Evid.-Based Complement. Altern. Med..

[51] Bai X., Zheng E., Tong L., Liu Y., Li X., Yang H., Jiang J., Chang Z., Yang H. (2024). Angong Niuhuang Wan inhibit ferroptosis on ischemic and hemorrhagic stroke by activating PPARγ/AKT/GPX4 pathway. J. Ethnopharmacol..

[52] Walter E., Gibson O.R. (2020). The efficacy of steroids in reducing morbidity and mortality from extreme hyperthermia and heatstroke—A systematic review. Pharmacol. Res. Perspect..

[53] Tang J., Deng P., Jiang Y., Tang Y., Chen B., Su L., Liu Z. (2013). Role of HMGB1 in propofol protection of rat intestinal epithelial cells injured by heat shock. Cell Biol. Int..

[54] Garcia C.K., Sheikh L.H., Iwaniec J.D., Robinson G.P., Berlet R.A., Mattingly A.J., Murray K.O., Laitano O., Clanton T.L. (2020). Effects of Ibuprofen during Exertional Heat Stroke in Mice. Med. Sci. Sports Exerc..

[55] Umemura Y., Ogura H., Matsuura H., Ebihara T., Shimizu K., Shimazu T. (2018). Bone marrow-derived mononuclear cell therapy can attenuate systemic inflammation in rat heatstroke. Scand. J. Trauma. Resusc. Emerg. Med..

[56] Chen S.H., Chang F.M., Chang H.K., Chen W.C., Huang K.F., Lin M.T. (2007). Human umbilical cord blood-derived CD34+ cells cause attenuation of multiorgan dysfunction during experimental heatstroke. Shock.

[57] Wang L., Deng Z., Zhao Y., Yuan R., Yang M., Zhang Y., Li Y., Liu Y., Zhou F., Kang H. (2021). Mesenchymal stem cells regulate activation of microglia cells to improve hippocampal injury of heat stroke rats. J. Therm. Biol..

[58] Chang C.P., Huang W.T., Cheng B.C., Hsu C.C., Lin M.T. (2007). The flavonoid baicalin protects against cerebrovascular dysfunction and brain inflammation in experimental heatstroke. Neuropharmacology.

[59] Li P., Shen T., Luo X., Yang J., Luo Z., Tan Y., He G., Wang Z., Yu X., Wang Y. (2021). Modulation of microglial phenotypes by dexmedetomidine through TREM2 reduces neuroinflammation in heatstroke. Sci. Rep..

[60] Xiao G., Yuan F., Geng Y., Qiu X., Liu Z., Lu J., Tang L., Zhang Y., Su L. (2015). Eicosapentaenoic acid enhances heatstroke-impaired intestinal epithelial barrier function in rats. Shock.

[61] Tsai H.Y., Hsu Y.J., Lu C.Y., Tsai M.C., Hung W.C., Chen P.C., Wang J.C., Hsu L.A., Yeh Y.H., Chu P. (2021). Pharmacological Activation Of Aldehyde Dehydrogenase 2 Protects Against Heatstroke-Induced Acute Lung Injury by Modulating Oxidative Stress and Endothelial Dysfunction. Front. Immunol..

[62] Tsai Y.C., Lam K.K., Peng Y.J., Lee Y.M., Yang C.Y., Tsai Y.J., Yen M.H., Cheng P.Y. (2016). Heat shock protein 70 and AMP-activated protein kinase contribute to 17-DMAG-dependent protection against heat stroke. J. Cell. Mol. Med..

[63] Hii H.P., Lo W.Z., Fu Y.H., Chen M.H., Shih C.C., Tsao C.M., Ka S.M., Chiu Y.L., Wu C.C., Shih C.C. (2022). Improvement in heat stress-induced multiple organ dysfunction and intestinal damage through protection of intestinal goblet cells from prostaglandin E1 analogue misoprostol. Life Sci..

[64] Varasteh S., Braber S., Kraneveld A.D., Garssen J., Fink-Gremmels J. (2018). L-Arginine supplementation prevents intestinal epithelial barrier breakdown under heat stress conditions by promoting nitric oxide synthesis. Nutr. Res..

[65] Lin X., Zhao T., Lin C.H., Zuo D., Ye Z., Lin S., Wen S., Liu L., Lin M.T., Chang C.P. (2018). Melatonin provides protection against heat stroke-induced myocardial injury in male rats. J. Pharm. Pharmacol..

[66] Lin X.J., Mei G.P., Liu J., Li Y.L., Zuo D., Liu S.J., Zhao T.B., Lin M.T. (2011). Therapeutic effects of melatonin on heatstroke-induced multiple organ dysfunction syndrome in rats. J. Pineal Res..

[67] Wu W.S., Chou M.T., Chao C.M., Chang C.K., Lin M.T., Chang C.P. (2012). Melatonin reduces acute lung inflammation, edema, and hemorrhage in heatstroke rats. Acta Pharmacol. Sin..

[68] Chen G.D., Fan H., Zhu J.H. (2019). Salidroside pretreatment protects against myocardial injury induced by heat stroke in mice. J. Int. Med. Res..

[69] Lin X., Lin C.H., Zhao T., Zuo D., Ye Z., Liu L., Lin M.T. (2017). Quercetin protects against heat stroke-induced myocardial injury in male rats: Antioxidative and antiinflammatory mechanisms. Chem. Biol. Interact..

[70] Zhao Y.-Q., Gao J.-T., Liu S.-H., Wu Y., Lin M.-T., Fan M. (2010). Geranylgeranylacetone preconditioning may attenuate heat-induced inflammation and multiorgan dysfunction in rats. J. Pharm. Pharmacol..

[71] Hall D.M., Buettner G.R., Oberley L.W., Xu L., Matthes R.D., Gisolfi C.V. (2001). Mechanisms of circulatory and intestinal barrier dysfunction during whole body hyperthermia. Am. J. Physiol. Heart Circ. Physiol..

[72] Chen F., Li H., Zhu G., Chen X., Tang Z. (2017). Sodium tanshinone IIA sulfonate improves inflammation, aortic endothelial cell apoptosis, disseminated intravascular coagulation and multiple organ damage in a rat heat stroke model. Mol. Med. Rep..

[73] Chang C.K., Chang C.P., Liu S.Y., Lin M.T. (2007). Oxidative stress and ischemic injuries in heat stroke. Prog. Brain Res..

[74] Mota R.A., Hernández-Espinosa D., Galbis-Martinez L., Ordoñez A., Miñano A., Parrilla P., Vicente V., Corral J., Yélamos J. (2008). Poly (ADP-ribose) polymerase-1 inhibition increases expression of heat shock proteins and attenuates heat stroke-induced liver injury. Crit. Care Med..

[75] Lin M.T., Kao T.Y., Jin Y.T., Chen C.F. (1995). Interleukin-1 receptor antagonist attenuates the heat stroke-induced neuronal damage by reducing the cerebral ischemia in rats. Brain Res. Bull..

[76] Tong H.S., Tang Y.Q., Chen Y., Yuan F.F., Liu Z.F., Peng N., Tang L.Q., Su L. (2013). HMGB1 activity inhibition alleviating liver injury in heatstroke. J. Trauma Acute Care Surg..

[77] Ohbe H., Isogai S., Jo T., Matsui H., Fushimi K., Yasunaga H. (2019). Treatment with Antithrombin or Thrombomodulin and Mortality from Heatstroke-Induced Disseminated Intravascular Coagulation: A Nationwide Observational Study. Semin. Thromb. Hemost..

[78] Li L., Tan H., Zou Z., Gong J., Zhou J., Peng N., Su L., Maegele M., Cai D., Gu Z. (2020). Preventing necroptosis by scavenging ROS production alleviates heat stress-induced intestinal injury. Int. J. Hyperth..

[79] Moon M., Huh E., Lee W., Song E.J., Hwang D.S., Lee T.H., Oh M.S. (2017). Coptidis Rhizoma prevents heat stress-induced brain damage and cognitive impairment in mice. Nutrients.

[80] Li L., Wang M., Chen J., Xu Z., Wang S., Xia X., Liu D., Wang S., Xie C., Wu J. (2021). Preventive Effects of Bacillus licheniformis on Heat Stroke in Rats by Sustaining Intestinal Barrier Function and Modulating Gut Microbiota. Front. Microbiol..

[81] Ren M.Q., Kazman J.B., Abraham P.A., Atias-Varon D., Heled Y., Deuster P.A. (2019). Gene expression profiling of humans under exertional heat stress: Comparisons between persons with and without exertional heat stroke. J. Therm. Biol..

[82] Durham W.J., Aracena-Parks P., Long C., Rossi A.E., Goonasekera S.A., Boncompagni S., Galvan D.L., Gilman C.P., Baker M.R., Shirokova N. (2008). RyR1 *S*-Nitrosylation Underlies Environmental Heat Stroke and Sudden Death in Y522S RyR1 Knockin Mice. Cell.

[83] Yamazawa T., Kobayashi T., Kurebayashi N., Konishi M., Noguchi S., Inoue T., Inoue Y.U., Nishino I., Mori S., Iinuma H. (2021). A novel RyR1-selective inhibitor prevents and rescues sudden death in mouse models of malignant hyperthermia and heat stroke. Nat. Commun..

[84] Protasi F., Paolini C., Dainese M. (2009). Calsequestrin-1: A new candidate gene for malignant hyperthermia and exertional/environmental heat stroke. Proc. J. Physiol..

